# Systemic Administration of Allogeneic Mesenchymal Stem Cells Does Not Halt Osteoporotic Bone Loss in Ovariectomized Rats

**DOI:** 10.1371/journal.pone.0163131

**Published:** 2016-10-06

**Authors:** Shuo Huang, Liangliang Xu, Yuxin Sun, Sien Lin, Weidong Gu, Yamei Liu, Jinfang Zhang, Lin Chen, Gang Li

**Affiliations:** 1 Department of Rehabilitation Medicine, Center of Bone Metabolism and Repair, State Key Laboratory of Trauma, Burns and Combined Injury, Trauma Center, Research Institute of Surgery, Daping Hospital, Third Military Medical University, Chongqing, PR China; 2 Department of Orthopaedics & Traumatology, Faculty of Medicine, The Chinese University of Hong Kong, Prince of Wales Hospital, Shatin, Hong Kong, PR China; 3 Stem Cells and Regenerative Medicine Laboratory, Li Ka Shing Institute of Health Sciences, The Chinese University of Hong Kong, Prince of Wales Hospital, Shatin, Hong Kong, PR China; 4 The CUHK-ACC Space Medicine Centre on Health Maintenance of Musculoskeletal System, The Chinese University of Hong Kong Shenzhen Research Institute, Shenzhen, PR China; 5 The Orthopaedic Research Laboratory, Changzhou Seventh People’s Hospital, Changzhou, Jiangshu Province, PR China; 6 College of Fundamental Medical Sciences, Guang Zhou University of Traditional Chinese Medicine, Guang Zhou, PR China; The University of Adelaide, AUSTRALIA

## Abstract

Mesenchymal stem cells (MSCs) have innate ability to self-renew and immunosuppressive functions, and differentiate into various cell types. They have become a promising cell source for treating many diseases, particular for bone regeneration. Osteoporosis is a common metabolic bone disorder with elevated systemic inflammation which in turn triggers enhanced bone loss. We hypothesize that systemic infusion of MSCs may suppress the elevated inflammation in the osteoporotic subjects and slow down bone loss. The current project was to address the following two questions: (1) Will a single dose systemic administration of allogenic MSCs have any effect on osteoporotic bone loss? (2) Will multiple administration of allogenic MSCs from single or multiple donors have similar effect on osteoporotic bone loss? 18 ovariectomized (OVX) rats were assigned into 3 groups: the PBS control group, MSCs group 1 (receiving 2x10^6^ GFP-MSCs at Day 10, 46, 91 from the same donor following OVX) and MSCs group 2 (receiving 2x10^6^ GFP-MSCs from three different donors at Day 10, 46, 91). Examinations included Micro-CT, serum analysis, mechanical testing, immunofluorescence staining and bone histomorphometry analysis. Results showed that BV/TV at Day 90, 135, BMD of TV and trabecular number at Day 135 in the PBS group were significantly higher than those in the MSCs group 2, whereas trabecular spacing at Day 90, 135 was significantly smaller than that in MSCs group 2. Mechanical testing data didn’t show significant difference among the three groups. In addition, the ELISA assay showed that level of Rantes in serum in MSCs group 2 was significantly higher than that of the PBS group, whereas IL-6 and IL-10 were significantly lower than those of the PBS group. Bone histomorphometry analysis showed that Oc.S/BS and Oc.N/BS in the PBS group were significant lower than those in MSCs group 2; Ob.S/BS and Ob.N/BS did not show significant difference among the three groups. The current study demonstrated that systemic administration of allogenic MSCs had no obvious effect on osteoporotic bone loss in OVX rats when using the cells from the same donor; and repeated injection of allogeneic MSCs from different donors might promote bone loss in OVX rats. These findings indicate that despite allogenic MSCs systemic infusion is safe, their administration alone may not be an effective mean for preventing osteoporotic bone loss.

## Introduction

Osteoporosis, a progressive systemic skeletal disease, is defined as a bone mineral density of 2.5 standard deviations or more below the average of young and healthy adults as measured by dual-energy X-ray absorptiometry [1]. Osteoporosis is characterized by a decrease in bone mass, bone mineral density (BMD) and microarchitecture deterioration of bone tissue, with a consequent increased risk of the fragility fracture which may lead to becoming bedridden with secondary complications or even a life threatening in the elderly [1, 2]. Osteoporosis commonly results from estrogen deficiency, characterized with inadequate bone formation, excessive bone resorption and failure to produce optimal bone mass and strength [3].

Current treatments for osteoporosis fall into 4 classes: (1) lifestyle modifications, such as increased physical activity, reduction of alcohol consumption and cessation of smoking. (2) Vitamin D and calcium supplementation, which is recommended as a baseline treatment in every patient with osteoporosis [3]; (3) Anti-resorptive drugs and bisphosphonates, which are most widely used due to high affinity for bone, long-term safety, inexpensive and effective for a broad spectrum of osteoporosis types [4]; (4) Anabolic drugs, which stimulate bone formation rather than preventing its loss. Parathyroid hormone 1–84 given by subcutaneous injection was most widely used. Although some of drugs may be effective, most have limitations and side-effects such as: osteonecrosis, esophageal irritation, acute-phase reaction, hypocalcaemia, renal toxic effects, thromboembolic disease etc. [3, 4]. Therefore, novel therapies are still needed.

Mesenchymal stem cells (MSCs), a kind of multipotent stem cells, have innate ability to self-renew and differentiate into various cell types such as chondrocytes, adipocytes, osteoblasts etc. when given proper stimulation [5, 6]. MSCs have been used in treatment of various diseases [7–10], and there are also many compelling evidences that MSCs can repair bone and related defects in animal models [11–13]. However, functions of MSCs are still considered controversial: Some people supported that MSCs mediate tissue repair through replacing damaged cells due to multi-lineage differentiation potential of MSCs [14, 15]. Our previous studies also showed that systematically transplanted allogeneic mesenchymal stem cells were able to differentiate into osteoblasts at the fracture site to directly contribute to the femoral fracture healing in rats. The others reported that MSCs mainly regulate the inflammation and immune cells to have immunomodulatory potentials in vitro and in vivo [16–18]. Le Blanc and his team showed that MSCs could suppress the proliferation of both CD4^+^ and CD8^+^ T cells [19]. MSCs also have the capacity to regulate the activity of macrophages, B cells and natural killer cells [20–23], and reduce inflammation by regulating the apoptosis of immune cells [24–27]. Therefore, the purpose of this study was to investigate whether repeated systemic injection of allogeneic MSCs from different donors could slow down osteoporotic bone loss.

## Materials and Methods

### Chemicals

The chemicals used were all purchased from Sigma-Aldrich (USA) except where specified.

### Animal details

All rats were housed in a designated government approved animal facility at The Chinese University of Hong Kong in according to The Chinese University of Hong Kong's animal experimental regulations. Animal surgery was approved by the Animal Experimentation Ethics Committee of the Chinese University of Hong Kong, and carried out under the animal licenses issued by the Hong Kong SAR Government. All surgeries were performed under anesthesia, and efforts were made to minimize the suffering of the animals. 18 Sprague—Dawley (SD) female rats (3-month old, body weight 250-320g) were subjected to ovariectomy (OVX). Three 8-week-old female GFP (Green fluorescent protein) rats (Japan SLC, Inc., Japan) were used as different cell donors. All the animals were sacrificed with an overdose of pentobarbital.

### Isolation and characterization of BM-MSCs

Isolation procedures were separately carried out on three GFP-rats avoiding cell contaminations with each other. The isolated GFP-MSCs were labeled as D1-MSCs, D2-MSCs and D3-MSCs. Briefly, the rat was kindly terminated, both femurs were dissected and stored on ice in phosphate buffered saline (PBS). Under the laminar flow in biological safety cabinet, two ends of the femurs were excised, and the marrow cavity was repeatedly flushed by 10ml Alpha complete culture medium (with 10% fetal bovine serum, 1% PSN). 10 ml of marrow suspension were layered over 5 ml Lymphoprep^™^ (STEMCELL Technologies Inc., Vancouver, Canada) in a 50ml centrifuge tube, then centrifuged at 800g for 20 minutes at room temperature. After centrifugation a distinct band containing mononuclear cells at the medium interface was formed, cells were then carefully removed from the interface. The harvested cells were diluted with 5 ml culture medium and centrifuged at 800g for 5 min. Then cells were cultured in a 100 mm cell culture dish (Corning Life Sciences, Product #353803) in the Alpha complete culture medium at 37°C with 5% CO^2^ and 95% humidity. At 80–90% confluence, cells were harvested with 2.5% trypsin and re-plated by splitting at a ratio of 1:3 into 75cm^2^ cell culture flasks (Corning Life Sciences, Product #430641). Mesenchymal stem cell markers CD44 (abcam, Cat. No. ab23396), CD90 (BD, Cat. No. 551401), endothelial cell marker CD31 (abcam, Cat. No. ab64543) and hematopoietic marker CD45 (Santa Cruz, Cat. No. sc-7324) were examined by flow cytometry. Briefly, 1 × 10^5^ harvested cells were stained with the Fluorescent Phycoerythrin (PE)-conjugated CD31, CD45, CD44, and CD90 for 1 hour at 4°C. The cells stained with PE-labeled IgG served as controls. The cells were pelleted, washed twice with PBS and resuspended in 0.5 ml stain buffer (BD Pharmingen, Franklin Lakes, NJ, USA) for flow cytometry analysis. WinMDI 2.9 software (The Scripps Research Institute, La Jolla, CA, USA) was used to create the histograms. In addition, these MSCs were also characterized by multi-lineage differentiation analysis. MSCs at passage 5 were used in this study.

### Animal surgery and experimental groups

The female rat was put under general anesthesia and sterile condition, a short dorsal midline skin incision was made halfway between the caudal edge of the ribcage and the base of the tail. Abdominal muscle wall incisions were made bilaterally to access to the peritoneal cavity, the ovary and the oviduct were taken out through the muscle wall incision. A sterile ligature is placed around the oviduct; the ovary was then excised through the oviduct near the ovary. The remaining tissue was moved back into the peritoneal cavity. The contralateral ovary was removed in a similar manner. At last the incisions were closed.

Following the surgery, the 18 rats were randomly assigned into 3 groups: (1) the PBS control group (PBS group): each rat was given 0.5ml PBS at Day 10, 46, 91 following OVX; (2) The MSCs injection group 1 (MSCs group 1): each rat was given 2x10^6^ D1-MSCs in 0.5ml PBS at Day 10, 46, 91 following OVX; (3) the MSCs injection group 2 (MSC group 2): each rat was given 2x10^6^ D1-MSCs in 0.5ml PBS at Day 10 following OVX, D2-MSCs at Day 46 and D3-MSCs at Day 91. Intra-cardiac injection guided by ultrasound imaging system (Vevo 770, VisualSonics Inc., USA) was employed in this experiment.

### The time-points for MSCs injection

For GFP-MSCs injection we designed 3 time points: at Day 10, 46, 91 following OVX. The first injection was set after 10 days following OVX to allow animal recovery from the surgery. Normally the rat osteoporosis formed after 3 months following ovariectomy, therefore the first two MSC treatments were used to investigate effects of systemic administration of allogeneic MSCs during the osteoporosis development period, and the last injection was used to test effects of allogeneic MSCs after osteoporosis formed.

### Micro-computer tomography (Micro CT) examination in vivo

All 18 rats were scanned by Scanco Medical Viva CT40 (Scanco Ltd, Switzerland) in vivo at Day 0, 45, 90, 135 following OVX. For image acquisition, the rat was under general anesthesia and placed in a custom-made plastic holder; a total of 150 two-dimensional (2D) micro-tomographic slices with an 18-μm slice increment covering a total range of 2.7 mm from the growth plate to the distal of the left tibia were then scanned. 80 sequential slices of 2D CT images with the final region of 1.44mm were selected, and the region of trabecular bone was set in each image. A low-pass Gaussian filter (Gauss Sigma = 0.8, Gauss Support = 1) was used to partly suppress the noise in the volumes. Threshold at 120–1000 were used for all samples in this study. Bone volume (BV), tissue volume (TV), BV/TV, mean volumetric bone mineral density (BMD) of BV, BMD of TV, trabecular number, trabecular thickness and trabecular spacing for each sample were recorded. 3D reconstruction images of the trabecular bone were also performed using the software provided. The data were divided by that of Day 0 (baseline) and expressed as the percentage.

### Blood collection and serum analysis

10ml blood from each rat was collected by cardiac puncture immediately after the animals were sacrificed at day 135 following OVX, the blood was allowed to clot by leaving it undisturbed at room temperature for 30 minutes, then centrifuged at 1000 g, 4°C for 15 minutes. Serum was carefully removed from upper layer by the pipette and stored at -80°C until analysis. The levels of IFNγ, TNFα, GM-CSF, Rantes, IL-1α, IL-1β, IL-2, IL-4, IL-6, IL-10, IL-12 and IL-13 in serum in rats (3 rats per group) were measured by using rat inflammatory cytokines Multi-Analyte ELISArray Kit (catalog no. MER-004A, QIAGEN). Absorbance (the readings at 450 nm subtracted readings at 570 nm) of each cytokine was read and compared across 3 groups.

### Four-point bending mechanical testing

After the blood collection, both femurs of all 18 rats were excised; muscles, soft tissues were carefully removed. Left femurs were temporarily stored at -80°C, and then were tested to failure with a constant displacement rate of 5 mm/min by four-point bending device (H25KS Hounsfield Test Equipment Ltd. Redhill, Surrey, UK). The left femurs were loaded in the anterior-posterior direction with the inner and outer span of the blades set as 8 and 20 mm respectively. The long axis of the femora was oriented perpendicular to the blades during the test [28]. After testing the load-displacement curves of the femurs were generated by the built-in software (QMAT Professional Material testing software. Hounsfield Test Equipment Ltd. Redhill, Surrey, UK); ultimate load to failure, energy absorbed to failure (The area under the load-displacement curves and is known as the toughness) [29] and the modulus of elasticity (E-Modulus, the slope of the stress-strain curve and is known as the tissue stiffness) [29] were recorded and analyzed by the software.

### Hematoxylin and Eosin staining

The right femurs were fixed in 4% buffered formalin for one day, and then decalcified with 9% formic acid for about three weeks. Attempts were made to standardize the sectioning at a mid-sagittal plane of each specimen by cutting the specimen in half (longitudinally in a sagittal plane) using a slicing blade. Samples were subjected to tissue processing and then embedded in paraffin. Thin sections (7 μm) were cut on a Rotary Microtome (HM 355S, Thermo Fisher Scientific, Inc., Germany) along the long axis of each femur in sagittal plane. Sections were mounted on the coated slides. Paraffin was removed by immersing the slides in Xylene 2 changes of 5 minutes at room temperature. Slides were then taken through graded ethanol and distilled water, then stained with Hematoxylin and Eosin (H&E), at last dehydrated and mounted.

### Immunofluorescent staining for GFP

Briefly, antigen retrieval was done by immersing deparaffinized sections into 10 mM of citrate buffer at 60°C for 20 min. Sections were blocked by 5% goat serum in 1% BSA for 20 min, then incubated with the rabbit anti-GFP antibody (1:300; Life Technologies) overnight at 4°C. Sections were washed with PBS for three times, then incubated with the goat anti-rabbit IgG-FITC (1:1000; Santa Cruz) for 60 min at room temperature in the dark; at last mounted by fluoroshield mounting Medium With DAPI (Dako). Slides were examined and images were taken using a fluorescent microscope (Zeiss-spot; Carl Zeiss MicroImaging GmbH, Jena, Thuringia, Germany).

### Bone histomorphometric analysis

H&E staining images were sent to the digitizing image analysis system (Osteometrics, Inc., USA) for quantitative bone histomorphometric analysis. Briefly, regions of interest were chosen from the proximal femoral growth plate to the proximal femoral metaphysis located between 0.5 and 3 mm distal to the growth plate—epiphyseal junction. Osteoblast surface per bone surface (Ob.S/BS), the osteoblast number per bone surface (Ob.N/BS), osteoclast surface per bone surface (Oc.S/BS) and the osteoclast number per bone surface (Oc.N/BS) were measured and calculated according to the manufacturer’s manuals.

### Statistical Analysis

All quantitative data were transferred to statistical spreadsheets and analyzed by a commercially available statistical program SPSS version 16.0 (IBM, USA), one-way analysis of variance (ANOVA) or one-way ANOVA with repeated measures were used for comparison of mean values with p <0.05 as considered statistical significance.

## Results

### Characterization of rat mesenchymal stem cells

**Fig 1:**
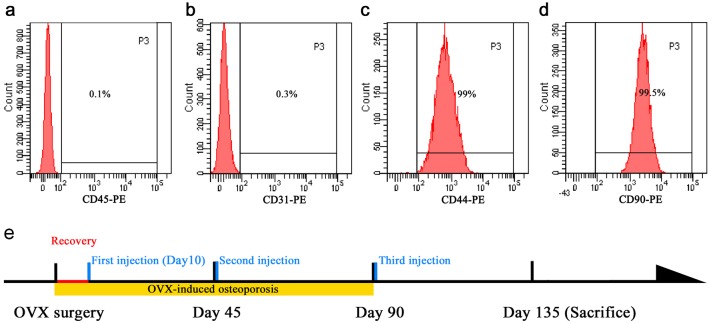
Immunophenotypic characterization of rat mesenchymal stem cells and time point setup. Flow cytometry analysis results confirmed that cells were negative for hematopoietic marker CD45 (a); Cells were negative for endothelial cell marker CD31(b); Isolated BM-MSCs were positive for mesenchymal stem cell markers CD44 and CD90 (c&d). (e) Time point setup. The timepoint that we injected GFP-MSCs was illustrated. The first injection was set after 10 days following OVX to allow animal recovery from the surgery. Normally the rat osteoporosis was induced by ovariectomy for 3 months, therefore the first two MSCs treatments were to investigate effects of injected MSCs during the osteoporosis development period, and the last injection was carried out to test effects of allogeneic MSCs after osteoporosis was formed.

In order to evaluate the effect of systemically administrated MSCs, as well as repeated injection of different donors-derived MSCs on osteoporosis, we isolated and cultured three different GFP-rats derived MSCs routinely, labeled as D1-MSCs, D2-MSCs and D3-MSCs respectively. All the MSCs were examined by flow cytometry, and all the cells showed CD markers for MSCs, one representative cell sample was shown in Fig 1a–1d.

### Micro CT analysis of the trabecular bone

**Fig 2:**
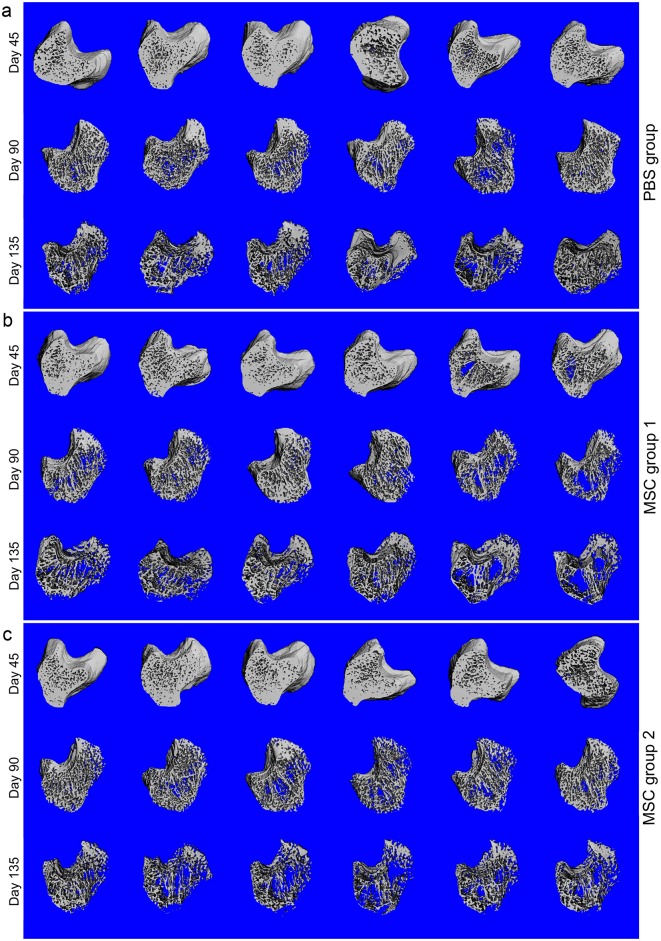
3D-Reconstruction images of the trabecular bone by Micro CT. All rats were scanned in vivo by Micro-CT at day 45, 90 and 135 following OVX; the morphology of trabecular bones showed that the trabecular bones were lost gradually with time after OVX in all samples, but no obvious morphological differences were found among the PBS group (a), MSC group 1 (b) and MSC group 2 (c).

The OVX model was established, the PBS or MSCs were systemically injected according to the Materials and Methods (Fig 1e). All rats were scanned in vivo by Micro CT at day 45, 90 and 135 following OVX. 3D-Reconstruction images of the trabecular bone (threshold at 120–1000) showed that after OVX the trabecular bone were lost gradually with time in the PBS group (Fig 2a), MSC 1 group (Fig 2b) and MSC 2 group (Fig 2c), but no obvious morphological difference was found among these three groups.

**Fig 3:**
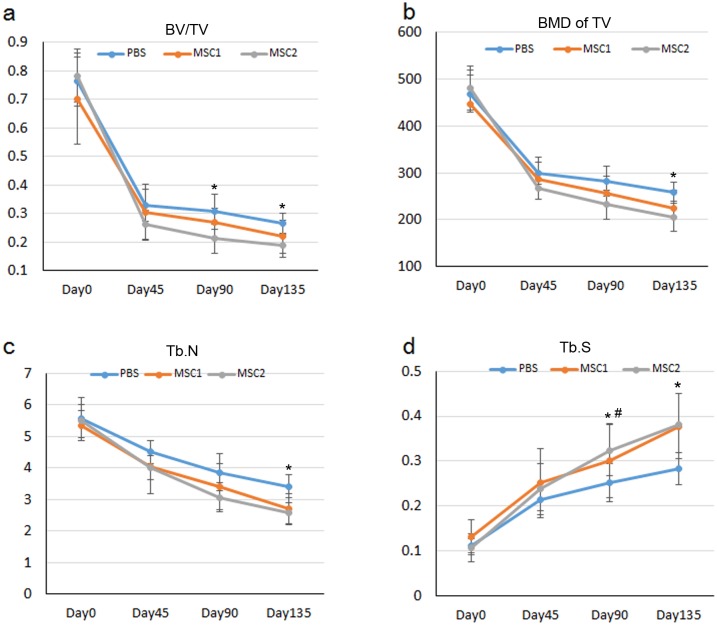
Micro-CT analysis of the trabecular bone. At Day 0, 45, 90, 135 following OVX, the trabecular bones were analyzed by Micro-CT. (a) BV/TV, (b) BMD of TV, (c) trabecular number and (d) Trabecular spacing were analyzed. Data was presented as mean ± SD (n = 8). Significant level was taken at p<0.05. *p<0.05, MSC2 compared with PBS group. #p<0.05, MSC2 compared with MSC1 group.

Interestingly, the quantitative analysis revealed that BV/TV (at Day 90, 135 following OVX), BMD of TV (at Day 135 following OVX) and trabecular number (at Day 135 following OVX) in the PBS group were significant higher than those in MSCs group 2 (Fig 3a–3c); Trabecular spacing (at Day 90, 135 following OVX) in MSCs 2 group were significant larger than that in the PBS control group (Fig 3d); but there was no significant difference between MSCs group 1 and the PBS control group, as well as the MSCs group 1 and MSCs group 2. Our finding showed that systemic injection of MSCs did not halt the progress of osteoporosis; on the other hand, the repeated injection of different donor-derived MSCs could lead to more rapid bone loss.

### Four-point Bending Mechanical Testing

To observe whether injection of MSCs affects the quality of bone, the femurs obtained from different groups were examined by mechanical testing. The result was shown in Table 1, demonstrating that the energy absorbed to failure (known as the toughness) in the MSCs group 2 was significantly lower than that in the MSCs group 1, but there was no significant difference between the MSCs group 1 and the PBS group, as well as the MSCs group 2 and PBS group. E-Modulus (known as the tissue stiffness) and ultimate load to failure did not show significant difference among these three groups. But all these three criteria showed a slight decrease trend in the MSCs group 2, suggesting that repeated injection of different donor-derived MSCs could affect the quality of bone.

**Table 1:** Four-point bending mechanical test of the fractured femur (n = 8).

	E-Modulus	Maximum Force	Energy Between
**PBS**	280.0±22.4	198.1±24.9	0.09±0.02
**MSC1**	266.8±40.5	202.9±14.8	0.11±0.02
**MSC2**	255.8±38.5	184.4±17.5	0.08±0.01*

Note:

*p<0.05, MSC2 compared with MSC1 group.

### Quantitative analysis of serum cytokine levels

**Fig 4:**
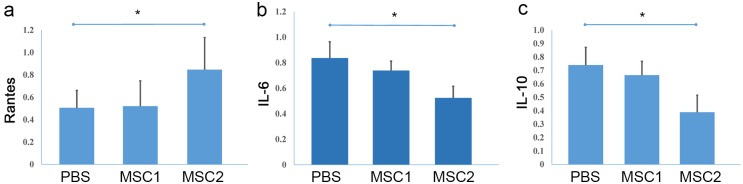
Quantitative analysis of cytokine levels in sera. Serum levels of (a) Rantes, (b) IL-6 and (c) IL-10 in SD rats of the three groups were evaluated at day 135 following OVX. Data was presented as mean ± SD (n = 8). Significant level was taken at p<0.05.

MSCs have been proved to have the ability to regulate the inflammation, we checked the levels of cytokines related to immune-response in the sera by ELISA assay. The result showed that the level of Rantes in serum in the MSCs group 2 was significantly higher than that in the PBS group, but there was no significant difference between the MSCs group 1 and the PBS control group, the MSCs group 1 and the MSCs group 2 (Fig 4a). The levels of IL-6 and IL-10 in sera in the MSCs group 2 were significantly lower than those in the PBS group, but there was no significant difference between the MSCs group 1 and the PBS control group, the MSCs group 1 and the MSCs group 2 (Fig 4b and 4c). Levels of GM-CSF, IFNγ, IL-1α, IL-1β, IL-2, IL-4, IL-12, IL-13 and TNFα in serum did not show significant differences among the three groups (S1 Fig).

### Bone histomorphometric analysis

**Fig 5:**
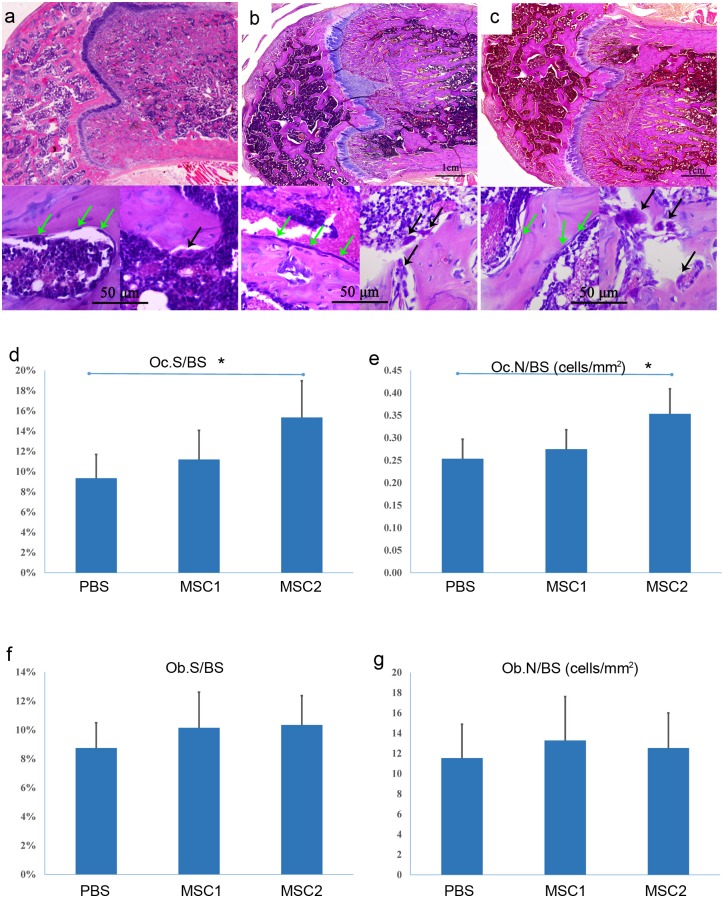
Bone histomorphometric analysis of the trabecular bones. H&E staining showed that trabecular bone had no obvious morphological difference among the PBS group (a), MSC group 1 (b) and MSC group 2 (c). Photomicrographs with higher magnification (×400) were attached on the bottom. Osteoblasts (green arrows) and osteoclasts (black arrows) were all observed in all three groups. (d&e) Quantitative analysis of the Oc.S/BS and Oc.N/BS in the three groups. Data was presented as mean ± SD (n = 8). Significant level was taken at p<0.05. (f&g) Quantitative analysis of the Ob.S/BS and Ob.N/BS in the three groups. Data was presented as mean ± SD (n = 8). Significant level was taken at p<0.05.

To check the involvement of bone formation and bone resorption during this process, bone histomorphometric analysis was conducted. Representative micrographs of paraffin sections with H&E staining showed that trabecular bones had no obvious morphological difference among the PBS (Fig 5a), MSC group 1 (Fig 5b) and MSC group 2 (Fig 5c). Quantitative analysis showed that Oc.S/BS and Oc.N/BS in the PBS group were significant lower than those in MSCs group 2 (p<0.05) (Fig 5d and 5e), whereas the Ob.S/BS and Ob.N/BS did not show significant difference among the three groups (p<0.05) (Fig 5f and 5g). In addition, we also detected the GFP-positive cells in the femurs by immunofluorescent staining with anti-GFP antibody, but no GFP positive cell was found in all three groups at day 135 following OVX (data not shown).

## Discussion

As we know that osteoporosis is a systemic bone disease due to imbalance between the bone resorption and bone formation which are regulated by a variety of growth factors and cytokines with elevated systemic inflammation which in turn triggers osteoclastogenesis and enhanced bone resorption (bone loss). Since MSCs are immunosuppressive and have been used to treat autoimmune diseases, we hypothesize that systemic infusion of MSCs may regulate or suppress the elevated inflammation in the osteoporosis subjects, hence slow down bone loss. This study investigated whether repeated injection of allogenic MSCs from the same or different donors could slow down the osteoporotic bone loss. The results demonstrated that there was no significant positive effect of allogenic MSCs systemic administration on osteoporotic bone loss; repeated injection of allogeneic MSCs from different donors even accelerated bone loss.

The underlying reasons that account for our findings could be: (1) Systemic administrated MSCs did not reach to the bone resorption or formation surfaces. Direct transplanted MSCs reach to the bone formation surface is of major importance in promoting bone formation. Min et al recently confirmed that transplanted MSCs without any modulations to enhance their bone homing potential have no significant positive effect on OVX rats [30]. However, when they developed a compound, a synthetic high-affinity with specific peptidomimetic ligand (LLP2A) against integrin α4β1 on the MSC surface, to a bisphosphonate (alendronate, Ale), and the compound was able to direct MSCs to the bone resorption surfaces in vivo, induced MSCs migration and osteogenic differentiation in vitro, and increased trabecular bone formation and bone mass [30]. Our current study did not use any specific preparation of MSCs, these MSCs may not be able to home to the bone resorption or bone formation surfaces efficiently, hence they did not enhance bone formation despite the repeated large dose system injection for 3 times. In the future, a special preparation to enhance the MSCs bone affinity shall be used to test the effects. (2) MSCs are immunosuppressive and systemic injection of allogenic MSCs has been reported to be immunosuppressive. Many papers reported that immunosuppressant agents could induce bone loss and osteoporosis [31–33]. In this study we tested cytokine levels in sera of the rats received MSCs injections, and the data showed that levels of 11 cytokines except Rantes seemed to be decreased in the MSCs group 2, but only levels of Rants, IL6 and IL10 showed statistical differences between the MSCs group 2 and the PBS control group (p<0.05). Rantes (CCl5), a chemotactic cytokine, is expressed and secreted by T cells, eosinophils, and basophils. Rantes is able to promote osteoblast migration via PI3K pathway and prevent apoptosis of osteoblasts [34, 35]. Rantes-deficient mice display decreased bone formation and increased osteoclastogenesis [36]. Interleukin-6 (IL-6), a pro-inflammatory and anti-inflammatory cytokine, is secreted by T cells and macrophages to stimulate immune response, and plays important roles in resistance against infection and bacterium [37]. IL-6 also can be secreted by osteoblasts to induce RANKL and stimulate osteoclastogenesis [38]. The higher level of Rantes and lower level of IL-6 in the MSC group 2 suggested reduced osteoclastogenesis and slowed down bone loss; but the opposite results seen on bone loss implied that these two cytokines may not play crucial roles in maintaining bone mass. Interleukin-10 (IL-10), an anti-inflammatory cytokine, has a potent inhibitory effect on osteoclastogenesis by suppressing NFATc1 activity to prevent differentiation of osteoclast progenitors into preosteoclasts at an early stage [39, 40]. IL-10 also plays an important role in bone metabolism in vitro and in vivo [40]. Clinical data analysis in Korean postmenopausal women suggested that variants of IL10 might play a role in the decreased BMD [41]. IL10-deficient mice developed osteoporosis [39]. Our results showed that the level of IL10 in the MSCs group 2 were significantly lower than that of the PBS group; hence this may be partially responsible for the accelerated bone loss seen in this group. Although intensive studies have done to investigate effects of many cytokines on BMD and/or osteoporosis, the cytokines involved are still largely unknown, whether IL10 is responsible for the induced osteoclastogenesis in this study still need to be confirmed. Repeated injection of allogenic MSCs from different donors may not be a good practice as this may trigger potential undesired immune effects. Taken together, the data suggested that after repeated infusion of allogenic MSCs from multiple donors, the inflammatory cytokines such as Rantes, IL-6 and IL-10 were significantly changed, suggesting that repeated infusion of allogenic MSCs may be pro-inflammatory and worsen the osteoporotic bone loss.

However, although there are important findings revealed by this study, there are limitations. First, we did not know the optimal timing and dose for MSCs delivery; and we did not use any carriers for MSCs delivery, these are the issues for consideration for any future investigations. Second, we don’t know if the changes of cytokines such as Rantes, IL10 and IL-6 are direct or indirect results of systemic allogenic MSCs administration, these need to be elucidated further. Despite the data in the present study were largely negative, the results provide useful insights for future studies of using allogenic MSCs for managing osteoporosis.

In conclusion, we demonstrated that there was no obvious adverse nor positive effects of systemic administration of allogenic MSCs on osteoporotic bone loss in the OVX rats. However, repeated injection of allogeneic MSCs from different donors may result a faster bone loss in the OVX rats, and shall be avoided. Preparations to enhance the allogenic MSCs bone affinity (specific targeting) or enhancing their survivability (including using MSCs from the same donor for repeated administration) shall be the future research directions for preventing osteoporotic bone loss using cell therapy approaches.

## Supporting Information

S1 Fig Quantitative analysis of cytokine levels in sera. Serum levels of GM-CSF, IFNγ, IL1α, IL1β, IL2, IL4, IL12, IL13 and TNFα in SD rats of the three groups were evaluated at day 135 following OVX. Data was presented as mean±SD (n = 8). Significant level was taken at p<0.05.
(PDF)
