# Correction: Systemic Administration of Allogeneic Mesenchymal Stem Cells Does Not Halt Osteoporotic Bone Loss in Ovariectomized Rats

**DOI:** 10.1371/journal.pone.0172462

**Published:** 2017-02-14

**Authors:** Shuo Huang, Liangliang Xu, Yuxin Sun, Sien Lin, Weidong Gu, Yamei Liu, Jinfang Zhang, Lin Chen, Gang Li

The following information is missing from the Funding section: Lin Chen (LC) was supported by a grant from the Chongqing City Basic and Frontier Research Project (CSTC2013jcyjC00009).

The following information is missing from the Acknowledgements section: The grant from the Chongqing City Basic and Frontier Research Project supported a crucial part of this study.

## References

[1] Huang S, Xu L, Sun Y, Lin S, Gu W, Liu Y, et al (2016) Systemic Administration of Allogeneic Mesenchymal Stem Cells Does Not Halt Osteoporotic Bone Loss in Ovariectomized Rats. PLoS ONE 11(10): e0163131 doi: 10.1371/journal.pone.0163131 27711227 10.1371/journal.pone.0163131 PMC5053541

