# Supplementary figures and images for: Systemic Administration of Allogeneic Mesenchymal Stem Cells Does Not Halt Osteoporotic Bone Loss in Ovariectomized Rats
Source: PLoS One. 2016 Oct 6;11(10):e0163131. doi: 10.1371/journal.pone.0163131 (PMC5053541; doi:10.1371/journal.pone.0163131)

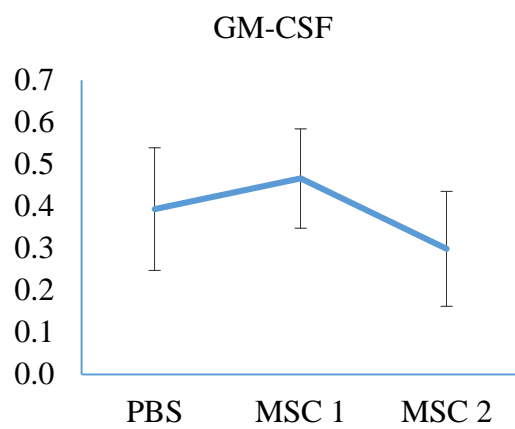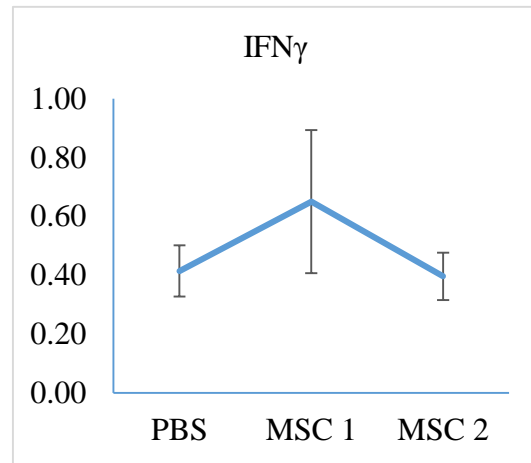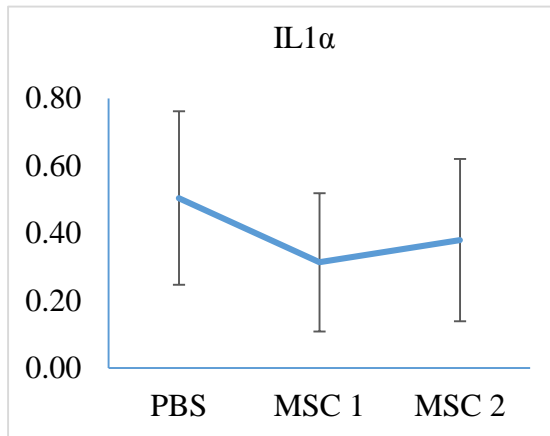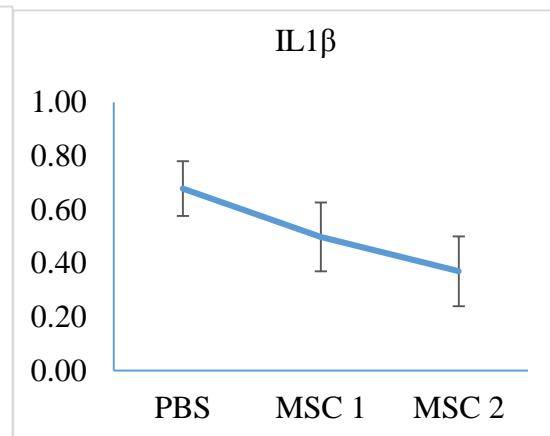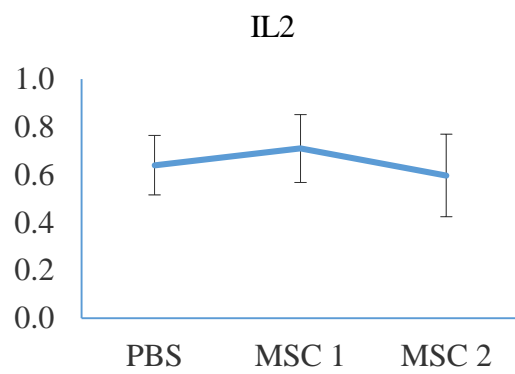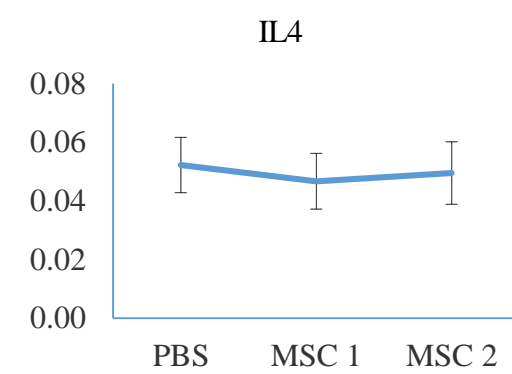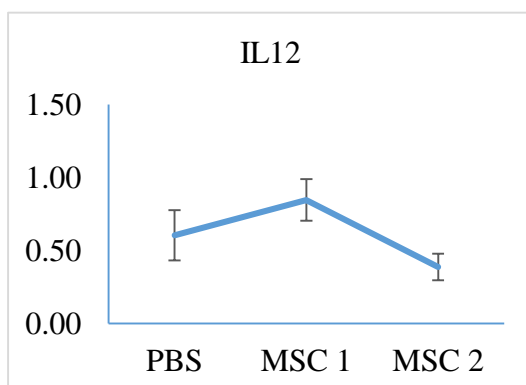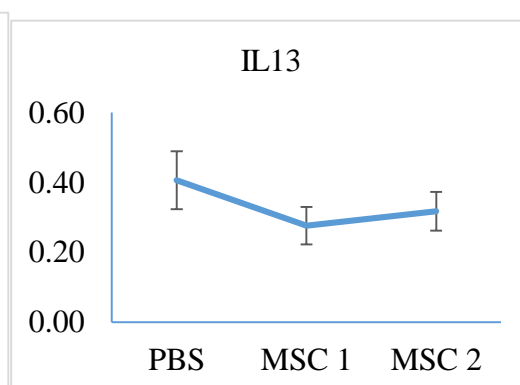

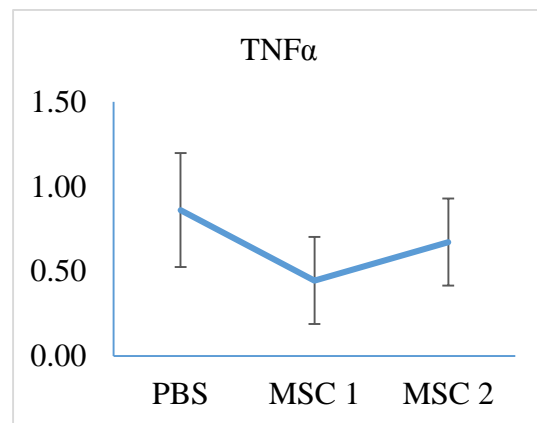

Supplement: S1 Fig — Serum levels of GM-CSF, IFNγ, IL1α, IL1β, IL2, IL4, IL12, IL13 and TNFα in SD rats of the three groups were evaluated at day 135 following OVX. Data was presented as mean±SD (n = 8). Significant level was taken at p<0.05. (PDF) [file pone.0163131.s001.pdf]
